# Satisfaction level with hearing aid in the daily life of Army healthcare system users

**DOI:** 10.1016/S1808-8694(15)31287-8

**Published:** 2015-10-20

**Authors:** Luciane R. Veiga, Álvaro R.C. Merlo, Sotero S. Mengue

**Affiliations:** 1 Master in Epidemiology, UFRGS; 2 Ph.D. in Sociology, Université Paris 7 – Denis Diderot; 3 Ph.D. in Pharmaceutical Sciences, UFRGS

**Keywords:** hearing aid, satisfaction, military, SADL questionnaire

## Abstract

The objective of the present study was to investigate satisfaction levels with hearing aids in daily life of Army Health System users, in addition to associated factors. Adults and seniors from 3^rd^ Military Area that had purchased hearing aids within the years 1998 and 2003 were selected to answer SADL (Satisfaction with Amplification in Daily Life) questionnaire. We excluded patients aged less than 18 years; those that had acquired hearing aid for less than 6 weeks, and patients with severe comprehension and expression limitation. The results showed that patients were considerably satisfied with the use of aids. There was lower satisfaction level with the negative factor subscale of SADL (Satisfaction with Amplification in Daily Life), especially in relation to telephone using. The factors that were associated with satisfaction were linked to the person and, mainly, to auditory rehabilitation. The data showed that, beyond the selection of the most technically appropriate hearing aid, it is highly important to follow auditory rehabilitation programs including home trials, guidance and counseling so that patients can have realistic expectations.

## INTRODUCTION

Satisfaction is the measurement of auditory rehabilitation outcome that represents the most comprehensive combination of factors required to define final outcomes, given that the variable of interest represents the point of view of the patient and it is not related only with performance of hearing aid (Cox and Alexander, 1999), depending exclusively on people's perceptions and attitudes (Hosford-Dunn and Halpern, 2000).

Since it involves judgments that reflect personal and subjective circumstances about expectations, needs and desires they do not allow objective measurements (Crow et al., 2002). This type of measurement of result is used to conduct surveys about effectiveness of treatment, serves as a basis for development of clinical practice guidelines and directs planning to compare, improve and standardize health care (Beck, 2000).

Despite technological advance of modern acoustic amplification systems, satisfaction of users is still a challenge to most audiologists and high rates of use cessation is still a problem for health services. In the United States, the rate of dissatisfied users of hearing aids has already reached 47%, out of which approximately 18% end up giving up auditory rehabilitation (Kochkin, 1996). In Brazil, these data are still unknown.

Hearing loss is one of the most devastating sensorial deficits because it impairs communication and leads to emotional, social and occupational sequelae. Hearing deterioration is normally a factor that announces the beginning of aging (Russo, 1999).

About 90% of the people aged over 80 years present hearing loss. As an expected consequence of the aging population, the number of candidates to wear a hearing aid will increase in upcoming years (Wisconsin Self Help for Hard of Hearing People Association, 2002).

In the services provided by the Military Medical Services of Porto Alegre, the Health Care Fund of the Army has high annual expenditure with acquisition of hearing aids to be prescribed its users. The cost of providing hearing aids to users, the importance of the success of aural rehabilitation in the life of subjects who have hearing loss and complexity of adapting acoustic amplification were motivations for the investigation of the result of aural rehabilitation.

Monitoring satisfaction levels is something important to assess clinical procedures, ensure the purposes of quality of services because satisfaction reflects the reality of health care results. Upon identifying the factors that contribute to satisfaction and upon trying to confirm these attributes in the involved processes, we reach the potential to have more effective results in healthcare services (Crow et al., 2002).

The purpose of the present study was to investigate the level of satisfaction with hearing aids in the daily life of users of Military Medical Services of Porto Alegre and to check associated factors.

## METHODS

**Figure 1:**
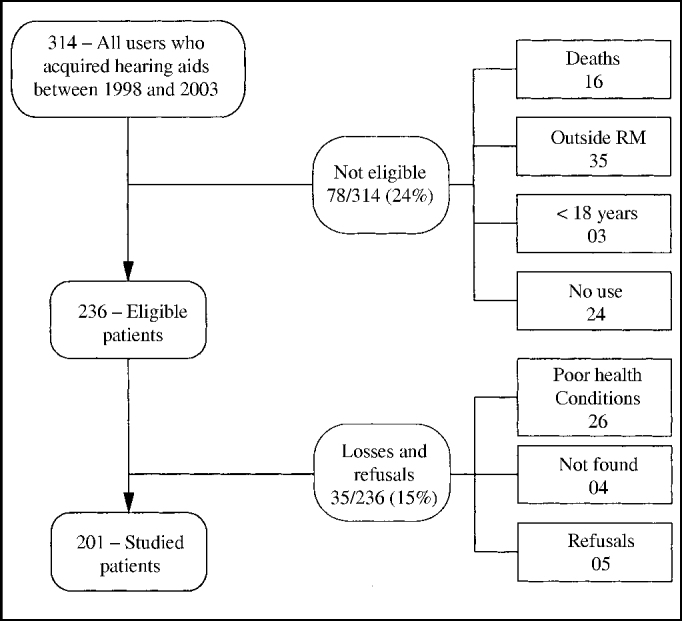
Studied population and satisfaction level with hearing aids among users of hearing aids acquired through FUSEX between 1998 and 2003.

We conducted a transversal study with 201 users of hearing aids acquired by the Health Care Fund of the Army (FUSEX), between years 1998 and 2003. The patients that acquired the hearing aids at that time but did not effectively use them (24 patients, which amounted to 7.6% of the total), did not join the study because the variable of interest that is part of the satisfaction rate depends on the point of view of the patient about systematic use of amplification (Cox and Alexander, 1999; Hosford-Dunn and Halpern, 2000) (Figure 1). We excluded from the study subjects who were aged less than 18 years, who had acquired hearing aids within less than 6 weeks, because satisfaction should be measured only after one month post-fitting to ensure reliability of results (Humes, 2002-a); people with severe limitation of understanding and expressing to respond the questionnaire, and those that did not agree to participate. The selection of patients started from the beneficiary charts of FUSEX and other information were collected from the database of Medical Service, Military Policy of Porto Alegre (PMPA).

The satisfaction rate with use of hearing aids in daily life was conducted using the questionnaire Satisfaction With Amplification in Daily Life – SADL, developed by Cox and Alexander (1999), with a sample of 257 subjects, mean age of 72 years, originated from an American medical center of the veterans, a community center for speech and hearing and a private clinic of hearing. SADL was validated by the authors in 2001. The instrument was prepared to assess satisfaction of people with the use of hearing aids, quantifying it by a four subscale score: Positive Effects (6 items associated with acoustic and psychological benefit), Services and Costs (3 items associated with professional competence, price of product and number of repairs), Negative Factors (3 items related to environmental noise amplification, presence of feedback, and use of telephone), and Personal Image (3 items related to esthetical factors and stigma of use of hearing aids) (total of 15 questions). The mean score of subscales, which are domains of satisfaction, results in the Overall Satisfaction score. The questions were answered with a 7-point scale of equal interval, corresponding to a categorical scale, from not at all to highly satisfied. For 11 questions, highly satisfied indicated complete satisfaction and was scored 7, whereas not at all indicated complete dissatisfaction and was scored 1. For the other 4 questions, the score was inverted, meaning that highly meant highly dissatisfied and not at all meant completely satisfied and scored 7.

To check whether possible factors were associated with satisfaction with use of hearing aids we also applied a questionnaire about the fitting process including the following subscales: Guidance Test, Hearing Center, Handling, Benefits, Limitations and Strategies of Communication. In addition, demographical and audiometric data were presented. The use of SADL in this population was anticipated with two independent translations to Portuguese, pre-tested with users of hearing aids from FUSEX, reviewed by the researcher concerning appropriateness of translation to the population, tested again with the modifications (replacement of words by more usual synonyms or omission of situations not contextualized for the target people, such as for example use of speaker phone) in users of hearing aids from FUSEX and hearing centers, submitted to opinion by specialists in the area, then sent for back-translation made by English native speaker, and tested with the final version in users of hearing aids of the center.

Patients were contacted by telephone and invited to come to the medical center. The questionnaire was self-applied to ensure the private nature that enhances validity of responses. Clarifications and guidance about the study and the questionnaire were provided by military layperson trained to do it. The total time required to answer the questionnaire was about 40 minutes. The main difficulty of very old patients was visual limitation to read the questions, and in some cases they used a magnifying glass or projection of questions on a large screen.

We conducted logistic regression and included the variables that presented statistically significance of at least 0.05 in bivariate analysis.

As to ethics, we used the informed consent term, signed in two copies based on the Regulating Guidelines and Rules for Healthcare Studies (Resolution nº 196/96). Project nº 2003125 was approved by the Ethics Research Committee, Federal University of Rio Grande do Sul (UFRGS). The authorization of the Military Healthcare Unit to conduct the study was published in the Internal Newsletter nº 074, dated May 9, 2003, of Military Medical Unit of Porto Alegre.

## RESULTS

The studied population was evenly distributed between female (49.3%) and male (51.7%) gender and educational level: 30.3% had completed elementary school, 32.3% had completed high school, and 37.3% had completed college education. There was predominance of race: 95% of the patients were Caucasians. As to military status, 73.1% of the main holders were officers and 51.2% of them were the actual patients. Mean age was 72 years. Most patients (60.2%) had family income below or equal to 6 minimum wages (Table 1).

**Table 1:** Baseline characteristics of participants in the study about satisfaction level with hearing aids among users that acquired them through FUSEX 3ª RM between 1998 and 2003.

Characteristics		Freq.	%
Gender			
	Female	99	49.3
	Male	102	51.7
Race			
	Caucasian	191	95.0
	Others	10	5.0
Education			
	Elementary School	61	30.3
	High school	65	32.3
	College level	75	37.3
Holder Status			
	Officer	147	73.1
	Non-Officer (private or civil)	54	26.9
Category of holder			
	Main holder	103	51.2
	Retired	55	27.4
	Dependent	43	21.4
Age (years)			
	< = 72	127	63.2
	> 72	74	36.8
Family income (minimum wage)			
	< = 6	121	60.2
	> 6	80	39.8

Upon checking validity of the translated questionnaire, we compared the overall satisfaction score of a categorical scale of simple items (highly dissatisfied, very dissatisfied, little dissatisfied, a little satisfied, satisfied, very satisfied, highly satisfied). We found strong correspondence of values, with mean of 5.05 for score of overall satisfaction of SADL and 5.16 for score of categorical scale of simple items. The values of percentiles were also quite close (Table 2).

**Table 2:** Comparison of scores of overall satisfaction with hearing aid use with scores of categorical scale of simple items in patients of FUSEX 3ª RM that acquired hearing aids between 1998 and 2003.

Satisfaction	Scores					
	Min.	25%	50%	75%	Máx.	Méd.
Overall SADL	2,5	4,5	5,1	5,7	7,0	5,05
Categorical scale	1,0	5,0	5,0	6,0	7,0	5,16

**Figure 2:**
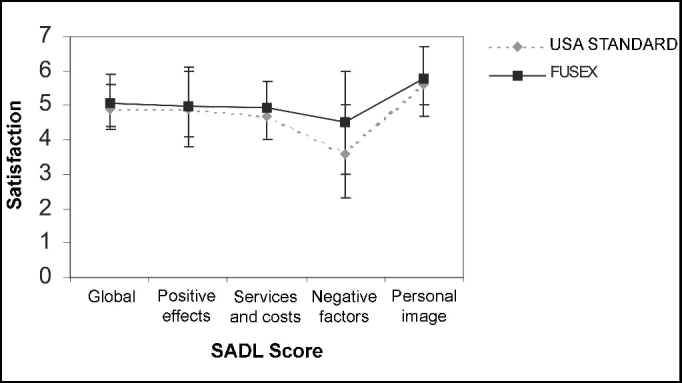
Comparative graph of overall satisfaction and subscale scores in the population of patients at FUSEX 3ª RM that acquired hearing aids between 1998 and 2003 and the standard of the American study (Cox and Alexander, 1999). Bars show percentile 20 and 80.

Regarding validity of questionnaire, upon comparing satisfaction with hearing aids in daily life between North-American standard (Cox and Alexander, 1999) and the present study, we detected similar results, with slightly better response among Brazilian subjects. Percentiles 20 and 80 were equivalent with small difference in subscale of negative factors. In both studies of subscale, what showed less satisfaction were the negative factors. A qualitative analysis in both populations showed SADL overall score in which subjects were considerably satisfied (Figure 2).

In the present study, mean scores of SADL and the respective correspondence of categorical scale were: overall score 5.05 (min. 2.5 and max. 7.0) –considerably satisfied; positive effects 4.99 (min. 1.2 and max. 7.0) –considerably satisfied; services and costs 4.94 (min. 2.0 and max. 7.0) –considerably satisfied; negative factors 4.5 (min. 1.0 and max. 7.0) –a little satisfied to considerably satisfied; and personal image 5.78 (min. 2.3 and max. 7.0) –very satisfied (Figure 2). Negative factors were the subscale that was farthest from the overall score, as a result of the low scores referring to hearing aids use on the phone. Personal image was also a subscale different from overall score as a result of the high scores to questions about use of hearing aids and perception of disability.

Upon analyzing factors associated with satisfaction with the use of hearing aids, it was defined high satisfaction as high score, referring to superior quartile, with cut-off point at 5.7 in the overall satisfaction scale SADL.

People-related factors that presented association with satisfaction in use of hearing aids were: absence of auditory disorders, absence of purely bilateral sensorineural components, and perception of good health.

Among the 25% patients with highest satisfaction score, there were 2.71 (CI 95%: 1.27 – 5.78) more chances of them being satisfied when they reported absence of hearing disorders (tinnitus and intolerance to loud sounds), 2.95 (CI 95%: 1.39 – 6.25) more chances of being satisfied when they did not have purely bilateral sensorineural loss; and 2.75 (CI 95%: 1.30 – 5.82) more chances of being satisfied when they reported perception of good health (Table 3).

**Table 3:** Proportion of subjects more satisfied with hearing aids in the upper quartile owing to people-related factors among users of FUSEX 3ª RM that acquired the hearing aids between 1998 and 2003.

	RC (IC 95%)
Absence of auditory damage	2.71 (1.27-5.,78)
Absence of bilateral sensorineural component	2.95 (1.39-6.25)
Perception of general good health status	2.75 (1.30-5.82)

Factors related to the process of auditory rehabilitation that presented association with satisfaction in the use of hearing aid were: having participated in the Auditory Rehabilitation Program and having received instructions on use and handling of hearing aids.

Among the 25% of patients with highest satisfaction rates, there were 2.88 (CI 95%: 1.37 – 6.04) more chances of them being satisfied when they had participated in the rehabilitation program, and 3.78 (CI 95%: 1.74 – 8.26) more chances of being satisfied when they had been instructed about the use and handling of hearing aids, regardless of having participated at the program (Table 4).

**Table 4:** Proportion of subjects more satisfied with hearing aids in the upper quartile owing to auditory rehabilitation factors, among users of FUSEX 3ª RM that acquired hearing aids between 1998 and 2003.

	RC (IC 95%)
Participation in Rehabilitation Program	2.88 (1.37-6.04)
Guidance on use and handling	3.78 (1.74-8.26)

## DISCUSSION

Results of overall score and subscales were equivalent to those of the original study (Cox and Alexander, 1999), reaching the same configuration in percentiles and in scales of higher or lower satisfaction levels.

The mean of SADL overall score showed that patients were considerably satisfied, but there was less satisfaction in relation to negative factors of hearing aids performance, especially telephone use, similarly to the population studied by Cox and Alexander (1999). The low score in negative factor subscale because of poor telephone use performance has already been reported in the literature as one of the main explanations for low satisfaction rates (Hosford-Dunn and Halpern, 2000). Northern (2000) has also reported in his studies that even though patients were satisfied, they had reported low improvement rate in telephone use. Telephone use is an auditory situation in which technological limitations of hearing aids are clearly evidenced. For this reason, guidance on use and handling of hearing aids should be reinforced by training telephone use and counseling should emphasize that difficulties are inevitable, so that the patients do not raise their expectations and end up being disappointed.

The other score that was highlighted was the personal image subscale, showing that normally patients did not associate use of hearing aids with image of disability. It is probably a response to the influence of human rights in the society. For the past decades, non-governmental associations and agencies have communicated the principles of equality and non-discrimination all over the world, and many have already included that in their Constitution. Thus, disabilities, among which hearing impairment, are more naturally seen and specific rights are assured to holders, trying to guarantee social insertion and reduction of prejudice. Hearing impaired subjects, as part of this society, have also changed their vision on their own hearing loss, which facilitates the acceptance of limitations without having the perception that they prevent normal functional life.

As to people-related factors, patients with fewer complaints of hearing loss (intolerance to loud noise and tinnitus) who did not have bilateral sensorineural loss had better results with hearing aids. Henderson et al. (1998) stated that even when wearing a hearing aid, patients with sensorineural loss and correlated symptoms (tinnitus and auditory recruitment) still have difficulty to understand acoustic information, especially speech sounds in noisy or reverberating environments. Non-ideal performance with hearing aids in these cases reflects in auditory processing disorders. Garstecki and Erler (1998) noticed that patients with sound intolerance would have deficit in their skills to benefit from the use of hearing aids. This finding is in accordance with the pathophysiological characteristics of hearing loss, since as previously demonstrated, if patients have sensorineural hearing loss, intolerance to loud sounds and tinnitus they are less benefited by acoustic amplification, and so it is expected that they would be less satisfied with the results obtained with hearing aids. It reinforces the importance of taking into account type of hearing loss and the presence of auditory damage in prescribing the characteristics of the hearing aids and guidance.

Another factor associated with high satisfaction level whose temporality was not reached in this study was the perception patients have of good general health status. To Crow et al. (2002), health status is a determining factor in satisfaction of patients seen by healthcare centers. Garstecki and Erler (1998) noticed better results in hearing aids use in patients that presented better health status. People in better general health status tend to be more active and consequently better prepared to face new situations, such as hearing aids fitting, that is, more prone to be satisfied. Conversely, satisfaction would reduce difficulty in communication and higher opportunities for socialization, reducing stress and allowing better general functional status. As we do not know which variable has more influence, we should at least be more attentive to general and psychosocial health aspects of patients when planning and conducting the auditory rehabilitation process.

As to factors related to process of auditory rehabilitation, this study showed that patients presented higher levels of satisfaction when they had participated in the Auditory Rehabilitation Program. In summary, the program developed by our center starts with assessment of global profile of the candidate to hearing aid fitting for planning the right focus on rehabilitation. The results of the audiological tests are explained to the patient and next we suggest that the patient should participate in one or more sessions on the topics: hearing loss and its implications, possibilities and limitations of the results of hearing aids use, and information about hearing aids. The patient that decides to continue in the process will take tests with different brands of hearing aids from vendors, followed by home trial period with the chosen model. Home trial, which is the result of the partnership between our center and hearing aids companies, provides free trial period of intra-aural hearing aids when this type of amplification system is prescribed. During the home trial period, the patient participates in counseling sessions, guidance and training on use and handling of hearing aids and communication strategies. If the patient acquired the hearing aids and does not require immediate follow up, he/she will be followed up within 3 months. If the hearing aid fitting is confirmed, we ask the patient to come back after one year or whenever needed.

If not properly adapted, we investigate the difficulties and provide new strategies, maintaining follow-up sessions up to complete fitting. It is important that the patient feels our support and that there is good interaction with the audiologist. Russo and Almeida (1995) emphasized that only by implementing a global rehabilitation program that supports adult hearing impaired people and their family members to deal with disadvantages and inabilities resultant from the deficit, in which the hearing aids are perceived as an integral part of the problem, there will be effective support to prevent social isolation and to reengage the patients in the world of verbal communication.

One of the main difficulties that hearing aid users have after acquisition is to turn occasional use into effective use. Therefore, professional intervention is longitudinal. No matter how well fitting has been made, following up the patients only at the acquisition moment is not enough. However, even though the literature in general advocates auditory rehabilitation programs, there are still no studies addressing their effectiveness. Moreover, data on rehabilitation programs should be carefully compared given that our program was developed by the researcher for her own use in daily clinical practice.

Data from previous studies together with these data suggest that guidance has a key role in satisfying hearing aids users. In the studied patients, counseling was made at the rehabilitation program and in the services provided by the hearing aid centers. For this reason, guidance was analyzed not only as part of the program, but also independently, finding a positive correlation with satisfaction, especially if related with generating realistic expectations. Many authors considered it a determining factor in hearing aid satisfaction, having close relation with perceptions about product performance (Crowley and Nabelek, 1996; Weinstein, 1997; Fabry, Jacobson and Newman, 2000; Crow et al., 2002). Even though, too high expectations of patients may result in disappointment and dissatisfaction (Cox and Alexander, 2000; Russo and Silveira, 2001). Since expectations are a factor necessarily perceived before hearing aid fitting, one important resource to reach satisfaction is to provide guidance for patients to be aware of limitations of the process. Moreover, we should let them know that they need persistence and patience to overcome the barriers of auditory rehabilitation and that with realistic expectations they will be ready to face them and reach a successful and satisfying fitting experience. Education is one more tool to make patients aware of their new condition, empowering them to become active, confident and independent agents to use and handle the hearing aids. Stika and Ross (2002) found that inappropriate guidance is a cause of dissatisfaction, and Sweetow (1999) and Russo and Silveira (2001) considered that a well-structured guidance and counseling program is essential to reach satisfaction.

Data showed that in addition to selecting the most technically appropriate hearing aid that has enough technology for each case, it is essential to reach high levels of satisfaction with hearing aids fitting with the implementation of an aural rehabilitation program. Findings suggest that such programs should count on home trial period and detailed instructions and guidance, aiming at transforming the patient into an active agent of the process, with knowledge and realistic expectations.

It is especially important to consider that public health services that provide hearing aids should realize that high cost with technological sophistication is not the only solution to prevent waste of cessation of use: support and educational programs are efficient allies.

The present study was conducted in a military center, whose environment is peculiar and the satisfaction profile has the specificities of the corporation. Thus, new research studies should be conducted to investigate the level of satisfaction of users of hearing aids in other populations to confirm these factors as key to satisfaction level of hearing aid users, as well as to clarify the effectiveness of auditory rehabilitation programs and the efficiency of different models.
